# Systems biology approach discovers comorbidity interaction of Parkinson's disease with psychiatric disorders utilizing brain transcriptome

**DOI:** 10.3389/fnmol.2023.1232805

**Published:** 2023-08-16

**Authors:** Md Asif Nashiry, Shauli Sarmin Sumi, Salem A. Alyami, Mohammad Ali Moni

**Affiliations:** ^1^Data Analytics, Northern Alberta Institute of Technology, Edmonton, AB, Canada; ^2^Computer Science and Engineering, Jashore University of Science and Technology, Jashore, Bangladesh; ^3^Mathematics and Statistics, Faculty of Science, Imam Mohammad Ibn Saud Islamic University (IMSIU), Riyadh, Saudi Arabia; ^4^Artificial Intelligence and Data Science, Faculty of Health and Behavioural Sciences, School of Health and Rehabilitation Sciences, The University of Queensland, Saint Lucia, QLD, Australia; ^5^Artificial Intelligence and Cyber Futures Institute, Charles Stuart University, Bathurst, NSW, Australia

**Keywords:** Parkinson's disease, psychiatric disorder, bipolar disorder, schizophrenia, differentially expressed genes

## Abstract

Several studies found that most patients with Parkinson's disorder (PD) appear to have psychiatric symptoms such as depression, anxiety, hallucination, delusion, and cognitive dysfunction. Therefore, recognizing these psychiatrically symptoms of PD patients is crucial for both symptomatic therapy and better knowledge of the pathophysiology of PD. In order to address this issue, we created a bioinformatics framework to determine the effects of PD mRNA expression on understanding its relationship with psychiatric symptoms in PD patients. We have discovered a significant overlap between the sets of differentially expressed genes from PD exposed tissue and psychiatric disordered tissues using RNA-seq datasets. We have chosen Bipolar disorder and Schizophrenia as psychiatric disorders in our study. A number of significant correlations between PD and the occurrence of psychiatric diseases were also found by gene set enrichment analysis, investigations of the protein-protein interaction network, gene regulatory network, and protein-chemical agent interaction network. We anticipate that the results of this pathogenetic study will provide crucial information for understanding the intricate relationship between PD and psychiatric diseases.

## 1. Introduction

The burden of neurological disorders is continuing to increase worldwide, and present data indicate Parkinson's disease (PD) has undergone the fastest growth in prevalence and disability among neurological disorders (Feigin et al., 2019). An estimated 10 million people worldwide are living with PD. Several studies predicted that the number of patients with PD would grow substantially in future decades. It was estimated that there would be nearly 5 million PD patients in China and about 1.2 million PD patients in the US by 2030 (Marras et al., 2018; Ou et al., 2021). Although PD is rare before the age of 50, this disease is increasingly common in each subsequent decade of life (Poewe et al., 2017). With the aging and increasing life span of the global population, age-related diseases, such as Parkinson's disease and Alzheimer's disease, are receiving much attention from researchers. PD is a neurodegenerative disorder of the central nervous system that leads to shaking, stiffness, and difficulty in walking, balance and coordination. People suffering from PD may have difficulties walking and speaking as the disease develops (Armstrong and Okun, 2020; Balestrino and Schapira, 2020; Jankovic and Tan, 2020). PD occurs when certain neurons in the basal ganglia region which produce dopamine, an important brain chemical, become impaired. The deficiency of dopamine in the brain causes abnormal brain activity which leads to impaired movement and other symptoms of PD (Höglinger et al., 2004; Triarhou, 2013). PD is often not recognized in its early stage because of the extended time between the first damage to the cells of the nervous system and the manifestation of clinical symptoms. Although the clinical features of PD are primarily connected with motor symptoms, PD is also associated with many non-motor The burden of neurological disorders is continuing to increase worldwide, and present data indicate PD has undergone the fastest growth in prevalence and disability among neurological disorders (Feigin et al., 2019). An estimated 10 million people worldwide are living with PD. Several studies predicted that the number of patients with PD would grow substantially in future decades. It was estimated that there would be nearly 5 million PD patients in China and about 1.2 million PD patients in the US by 2030 (Marras et al., 2018; Ou et al., 2021). Although PD is rare before the age of 50, this disease is increasingly common in each subsequent decade of life (Poewe et al., 2017). With the aging and increasing life span of the global population, age-related diseases, such as Parkinson's disease and Alzheimer's disease, are receiving much attention from researchers. PD is a neurodegenerative disorder of the central nervous system that leads to shaking, stiffness, and difficulty in walking, balance and coordination. People suffering from PD may have difficulties walking and speaking as the disease develops (Armstrong and Okun, 2020; Balestrino and Schapira, 2020; Jankovic and Tan, 2020). PD occurs when certain neurons in the basal ganglia region which produce dopamine, an important brain chemical, become impaired. The deficiency of dopamine in the brain causes abnormal brain activity which leads to impaired movement and other symptoms of PD (Höglinger et al., 2004; Triarhou, 2013). PD is often not recognized in its early stage because of the extended time between the first damage to the cells of the nervous system and the manifestation of clinical symptoms. Although the clinical features of PD are primarily connected with motor symptoms, PD is also associated with many non-motor symptoms (Aarsland et al., 2001; Schrag, 2004). For instance, Li et. al., studied the data of patients who were admitted to the hospitals for psychiatric disorders and PD during the period of 1987– 2001 (Li et al., 2008). One of the major findings of this study is that psychiatric disorder is an appreciable risk factor for the development of PD.

The impact of psychiatric disorder early in life on the subsequent risk of Parkinson's disease and its clinical manifestations remains unknown. Psychiatric diseases, including anxiety, depression, dementia, and schizophrenias, have been found as risk factors for Parkinson's disease in several investigations (Lin et al., 2014; Grover et al., 2015). In this work, we have chosen two psychiatric disorders, Bipolar disorder and Schizophrenia, in order to find the genetic relevance between PD and psychiatric disorder. More than 1% of the world's population suffers from bipolar disorder (BD). BD is one of the biggest causes of disability in young people, resulting in cognitive and functional impairment as well as an increased risk of death, notably suicide (Grande et al., 2016). hlBD is a psychiatric condition that can be characterized by recurrent episodes of elevated mood and depression as well as fluctuations in activities levels (Miklowitz and Johnson, 2008; Anderson et al., 2012). BD causes changes in one's mood, energy, and ability which impacts in performing regular activity. People with bipolar disorder endure extreme emotional states known as mood episodes which can be either manic/hypomanic (abnormally happy or angry mood) or depressive (sad mood) (Belmaker, 2004). Schizophrenia (SCZ) is a chronic psychiatric disorder that is associated with symptoms such as delusions, hallucinations, disorganized speech, and trouble with thinking (Van Os et al., 2010; McCutcheon et al., 2020). When the condition is active, the person may have episodes in which s/he is unable to distinguish between real and unreal events. Individuals with SCZ can have experiences such as hallucinations, delusions, and profoundly abnormal thought and behavior, which can make it difficult to function on a regular basis.

BD and SCZ are highly heritable disorders that have similar clinical characteristics (Ohi et al., 2020). In this work, we have used mRNA expression analysis in order to understand the complex interaction between PD and psychiatric disorders. We have used a statistical method to determine differentially expressed genes (DEGs) and used the shared DEGs to determine protein-protein interaction (PPI), gene regulatory networks (GRN) and protein-chemical interactions. We have also performed gene ontology and pathway analyses to determine significant regulatory checkpoints related to disease complications. The findings of this work may lead to the process of determining the significant therapeutic target to fight against the complicated relationship between PD and psychiatric illnesses.

## 2. Methods and materials

### 2.1. Overview of the workflow

Bioinformatics and network based approaches provide insight that can be used to identify and analyze the comorbidity complexities (Moni et al., 2019; Nashiry A. et al., 2021; Nashiry M. A. et al., 2021; Islam et al., 2022). At the initial stage of this analysis, the datasets required in this work have been identified and collected. We used RNA-Seq gene expression datasets for the disorders we were interested in. The differentially expressed genes from each of the datasets have been identified using gene expression analysis. Next, it has been determined which DEGs are shared by PD and the two psychiatric illnesses. These common DEGs are utilized to find enriched cell signaling pathways and functional Gene Ontology (GO) terms as well as to perform gene set enrichment analysis to determine their protein-protein interactions. Next, two different types of gene regulatory networks–DEGs-miRNAs (micro-RNA) network and DEGs-TF (transcription factors) network–are found using the same collection of common DEGs shared by PD and the psych. Additionally, interactions between proteins and chemical compounds have been discovered for the prevalent DEGs.

### 2.2. Gene expression datasets

In this study, we gathered RNA-Seq datasets from the National Center for Biotechnology Information's (NCBI) Gene Expression Omnibus (GEO) (Barrett et al., 2012). In this work, the dataset for Parkinson's disease with the GEO accession number GSE90514 is taken into consideration. In GSE90514, fibroblasts from four people with Parkinson's disease and four healthy controls were cultured, and whole RNA sequencing was used to examine the transcriptome profile. For schizophrenia, the dataset with accession number GSE63738 is taken into consideration. Using RNA sequencing, the global transcription of neural progenitor cells (NPCs) produced from human induced pluripotent stem cells (hiPSCs) from six SCZ patients and six controls is compared in GSE63738. The brain transcriptome in bipolar disorder is represented by the dataset (GSE53239) for BD. The brain transcriptomes of six people with BD and six healthy controls were examined using RNA-sequencing.

### 2.3. Identification of differentially expressed genes

On the datasets, we have run a number of statistical processes to identify the differentially expressed genes (DEGs). Statistical analyses for determining differentially expressed genes have been carried out using the limma (Linear Models for Microarray Analysis) R package (Ritchie et al., 2015). Additionally, a fair balance between the identification of statistically significant genes and the restriction of false positives is achieved by using the Benjamini-Hochberg false discovery rate approach. Genes that have an absolute value of log2 fold change ≥ 1 and an adjusted *p* < 0.05 are regarded as DEGs in this study. We have also identified the DEGs that are common between these three diseases.

### 2.4. Protein-protein interaction analysis

Using the DEGs that are shared by PD and two other psychiatric diseases, we have created protein-protein interaction (PPI) networks. PPI analysis sheds light on the relationship of the proteins. Using STRING (Szklarczyk et al., 2018), which is a protein interactome database, we have identified the protein network. The Markov cluster technique has been utilized to identify gene clusters (MCL).

### 2.5. Function enrichment analysis

Using the common DEGs, we conducted a functional enrichment analysis. We have used Enrichr (Kuleshov et al., 2016) in this analysis. The functional enrichment analysis found a number of functional Gene Ontology (GO) terms and enriched cell signaling pathways that demonstrate the biological importance of the discovered DEGs. The signaling pathways from six libraries, including BioPlanet, BioCarta, KEGG, Panther, Reactome, and WikiPathways, have been combined in Enrichr. Only the important pathways for which the adjusted p-value is less than 0.05 are taken into consideration after the duplicate pathways have been eliminated. We used the GO biological process (2018) dataset in Enrichr to analyse functional GO terms, and we identified the relevant GO terms using an adjusted *p* 0.05.

### 2.6. Gene regulatory networks analysis

Gene regulatory networks (GRN) analysis includes the discovery of DEG-miRNA (micro RNA) and transcription factor (TF)-DEG interaction networks. The shared dysregulated genes are used to identify DEG-miRNA and TF-DEG networks using Network Analyst platform (Xia and Hancock, 2015). TarBase (Vergoulis et al., 2011) and miRTarBase (Huang et al., 2019) databases are used for discovering DEG-miRNA interaction networks. JASPAR (Fornes et al., 2019) database is used to identify TF-DEG interaction network. The networks are filtered with the betweenness value of 500.

### 2.7. Protein-chemical compound analysis

The chemical agents responsible for the interaction of proteins in comorbidity are identified by the protein-chemical compound analysis. In this investigation, we used the enriched gene (common DEGs) that PD and psychiatric diseases share to identify protein-chemical interactions. Utilizing the Comparative Toxicogenomics Database (Davis AP, 2018), we identified protein-chemical interactions using Network Analyst (Xia and Hancock, 2015).

## 3. Result

### 3.1. Shared DEGs identify genetic relevance between PD and psychiatric disorders

**Figure 1:**
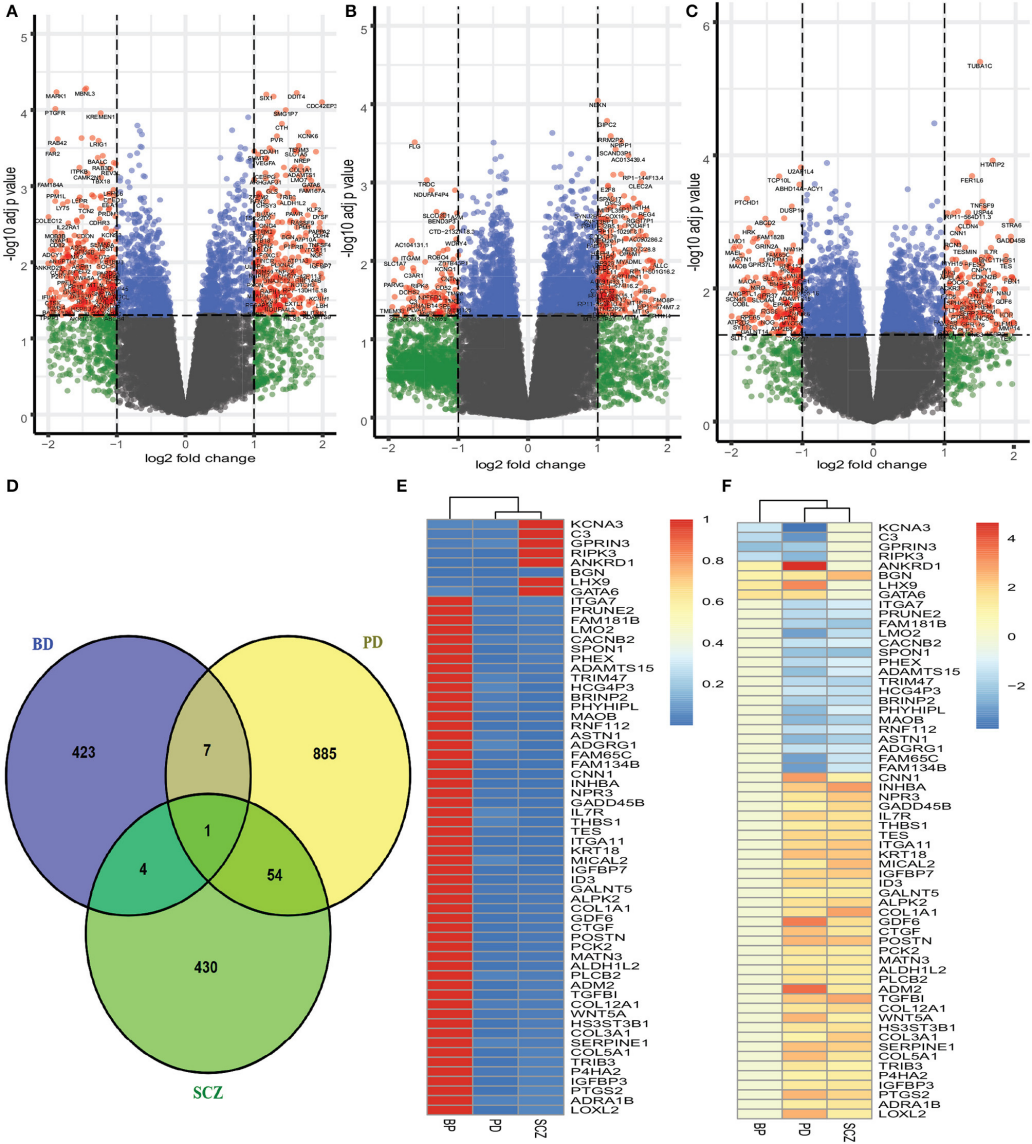
Red dots presented in volcano plots as shown in **(A–C)** represent the significant DEGs (differentially expressed genes) of PD, BD, and SCZ, respectively. The criteria chosen to be considered as DEGs are (i) absolute value of *log*_2_ *fold change* ≥1 (ii) *adjusted p-value* < 0.05. The Venn diagram in **(D)** shows the number of DEGs and common DEGs among the disorders including PD. Heatmaps show the relationships among common DEGs of different disorders based on **(E)** *adjusted p-value* and **(F)** *log*_2_ *fold change*.

We have analyzed the fibroblasts of PD patients and identified 947 genes that are differentially expressed as compared to healthy control. We have found 500 DEGs as down regulated among 947 DEGs, and the rest of the DEGs are up regulated. As we analyzed the brain trainscriptome of BD patients and healthy controls, our analysis discovers 177 down regulated and 258 up regulated DEGs (435 DEGs in total). Similarly, we have identified 489 DEGs (253 down regulated and 236 up regulated) in the case of SCZ. The volcano plots presented in Figure 1 show the DEGs for Parkinson's disease and two psychiatric disorders with the red dots. The number of shared DEGs between PD and two psychiatric disorders is presented in the Venn diagram shown in Figure 1D. The number of shared genes between PD and BD is 8 as is seen from the Venn diagram. The shared genes include KCNA3, C3, GPRIN3, RIPK3, ANKRD1, BGN, LHX9, and GATA6. PD shares 55 dysregulated genes with SCZ. These shared genes can be seen from the heatmaps in Figure 1. We have identified one gene, BGN, which is shared by all three disorders. In addition, we have found 5 dysregulated genes shared by BD and SCZ. The DEGs shared between PD and psychiatric disorders and their relationships from the perspective of *adjusted p-value* and *log*_2_ *fold-change* are presented in the heatmaps shown in Figures 1E, F, respectively.

### 3.2. Protein-protein interaction analysis shows the hub proteins and the interaction among proteins

**Figure 2:**
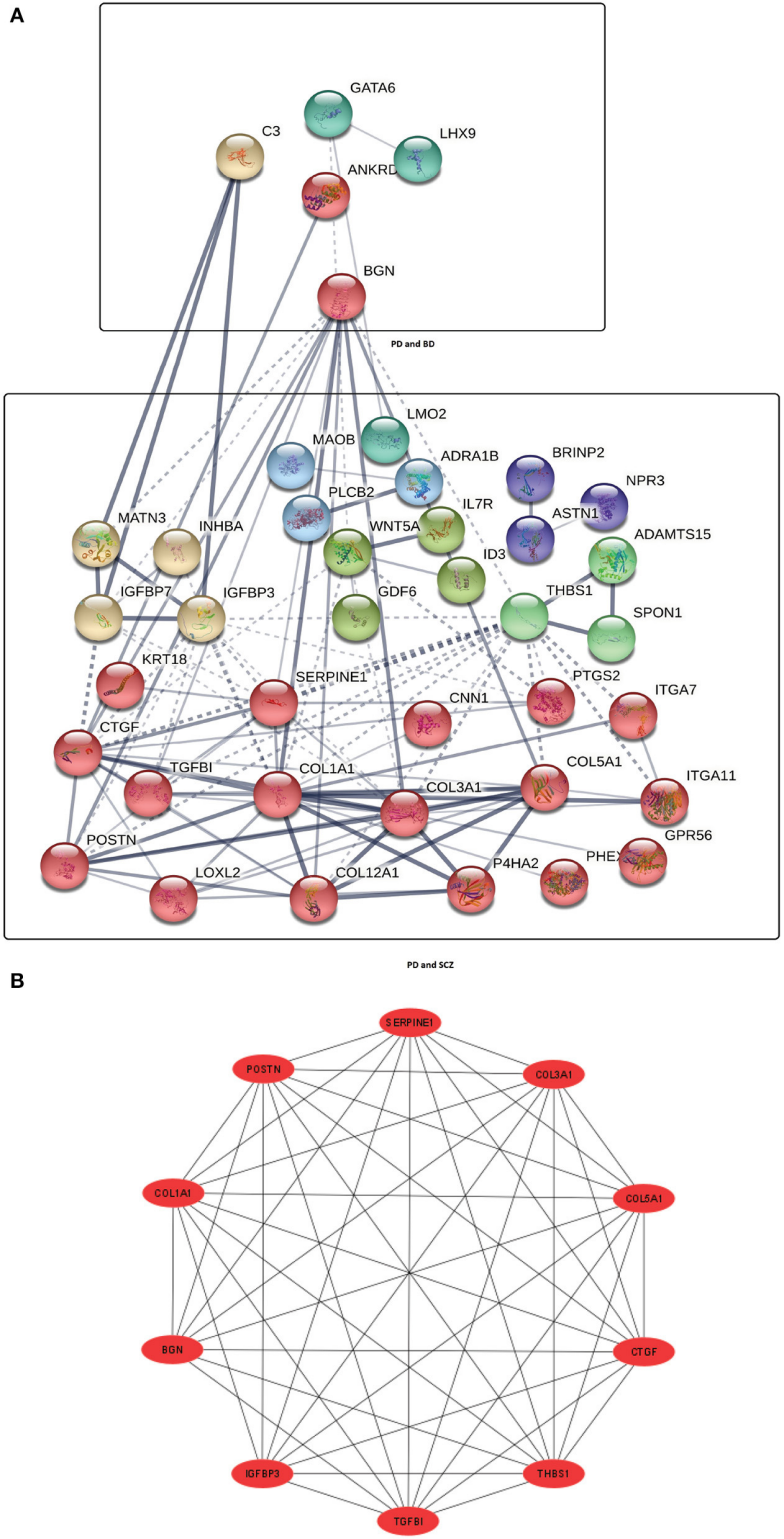
**(A)** Protein-protein interaction network of common DEGs in PD and psychiatric disorders. Square boxes represent the proteins shared by PD and the corresponding disease. For example, the top box represents the proteins that are common in the interaction between PD and BD. Proteins having several edges are highly expressed. **(B)** Hub protein network shows 10 significant hub proteins based on the number of interactions.

Next, We have employed protein-protein interaction analysis with the shared DEGs between PD and psychiatric disorders. The common DEGs between the three disorders are used to construct the protein-protein interaction network shown in Figure 2A. The rectangle boxes in the figure show the proteins involved in each of the psychiatric disorders and PD. The edges show the interaction among the proteins, and the thickness between the edges indicates the strength of the relation among the proteins. The proteins with several connecting edges can be identified as hub proteins (Ekman et al., 2006). Figure 2B shows a network of hub proteins. The network has been generated using Cytohubba (Chin et al., 2014) package of Cytoscape. The hub protein network displays the interaction of the hub proteins among each other.

### 3.3. Functional enrichment analysis identifies significant cell signaling pathways and gene ontology terms

**Figure 3:**
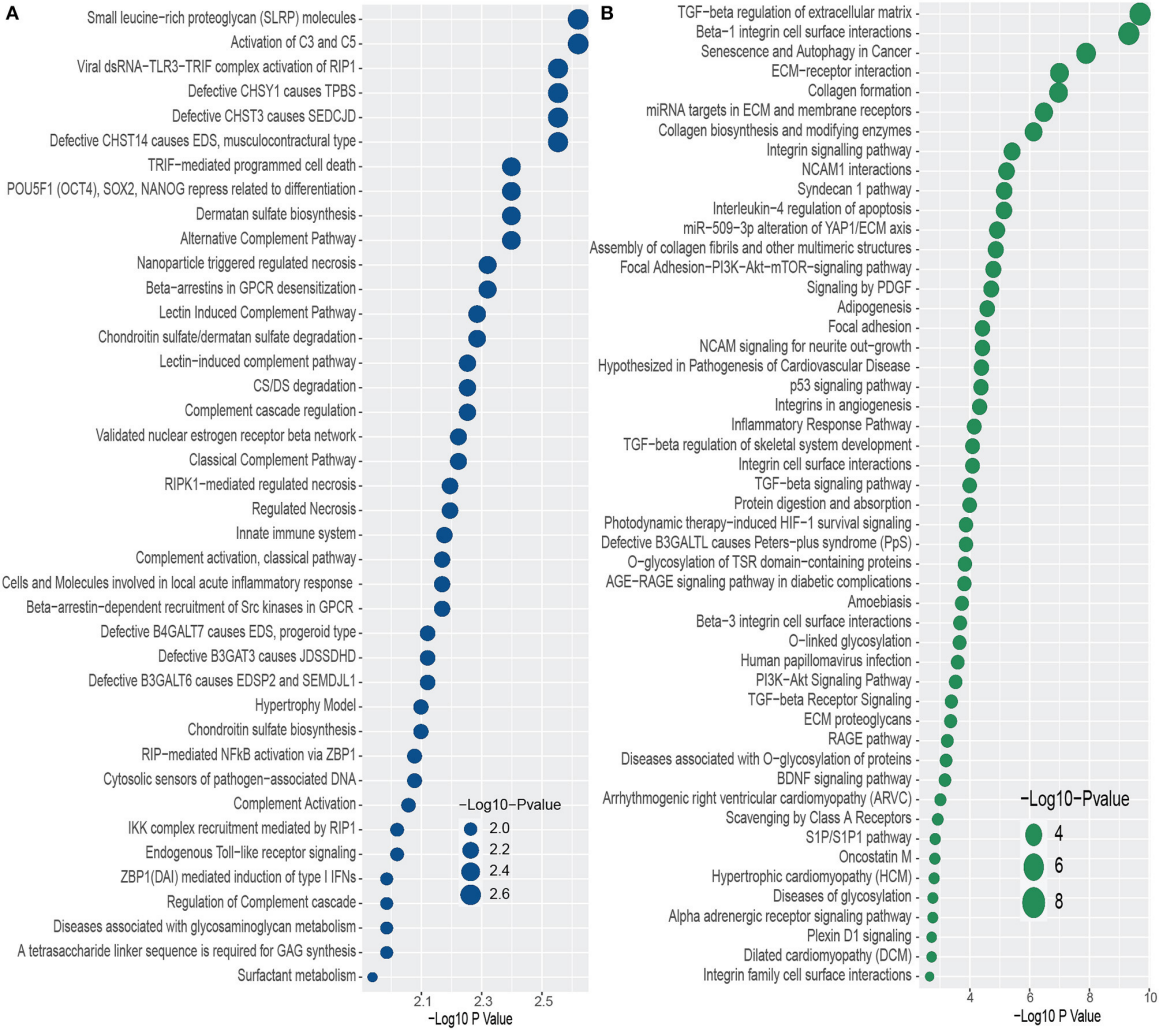
Top 40 cell signaling pathways based on the adjusted *p*-values between PD and psychiatric disorders. The pathways have been formed by combining the DEGs that are common in psychiatric disorder and PD. **(A, B)** Show the pathways for BD and SCZ, respectively.

We conducted functional enrichment analysis to identify significantly enriched cell signaling pathways and functional GO terms. In enrichment analysis, we have used Enrichr (Kuleshov et al., 2016). In this analysis, we have combined all the DEGs that have been discovered in the interaction of PD with psychiatric diseases and merged all the pathways from six pathway databases collected from Enrichr (Kuleshov et al., 2016) libraries i.e. BioPlanet, BioCarta, KEGG, Panther, Reactome, and WikiPathways. We have considered the pathways for which *adjusted p-value* is less than 0.05, and plotted the top 40 pathways for each of the psychiatric diseases in Figure 3. The pathways with higher logarithmic *adjusted p value* are considered significantly enriched. For example, the Small leucine-rich proteoglycan molecules and the Activation of C3 and C5 pathways are the most significantly enriched pathways in the interaction between PD and BD as observed from Figure 3A. We have also found the family of complement pathways which include Classical complement pathway, Lectin-induced complement pathway, and the alternative complement pathway. In addition, Viral dsRNA-TLR3-TRIF complex activation of RIP1, Defective CHSY1 causes TPBS, and the family of sulfotransferase pathways are also strongly enriched in the interaction between PD and BD.

In addition, we have found several pathways from the family of Galactosyltransferases such as defective B4GALT7 causes EDS, progeroid type, Defective B3GAT3 causes JDSSDHD, Defective B3GALT6 causes EDSP2 and SEMDJL1. In the case of PD-SCZ interaction, we have identified TGF–beta regulation of extracellular matrix, Beta–1 integrin cell surface interactions, and senescence and autophagy in Cancer pathways are the top three enriched cell signaling pathways. We have observed several TGF related pathways in the relationship between PD and SCZ. We have discovered several pathways related to cardiomyopathies such as arrhythmogenic right ventricular cardiomyopathy (ARVC), hypertrophic cardiomyopathy (HCM), and dilated cardiomyopathy (DCM). Other pathways include glycosylation related pathways, RAGE pathway, Signaling by PDGF, Focal adhesion, NCAM1 interactions, Interleukin–4 regulation of apoptosis, to name a few. Similar to the case of PD-BD interaction, we have found glucosyltransferase pathways such as defective B3GALTL causes Peters–plus syndrome (PpS) in one of the highly enriched pathways in PD-SCZ interaction.

**Figure 4:**
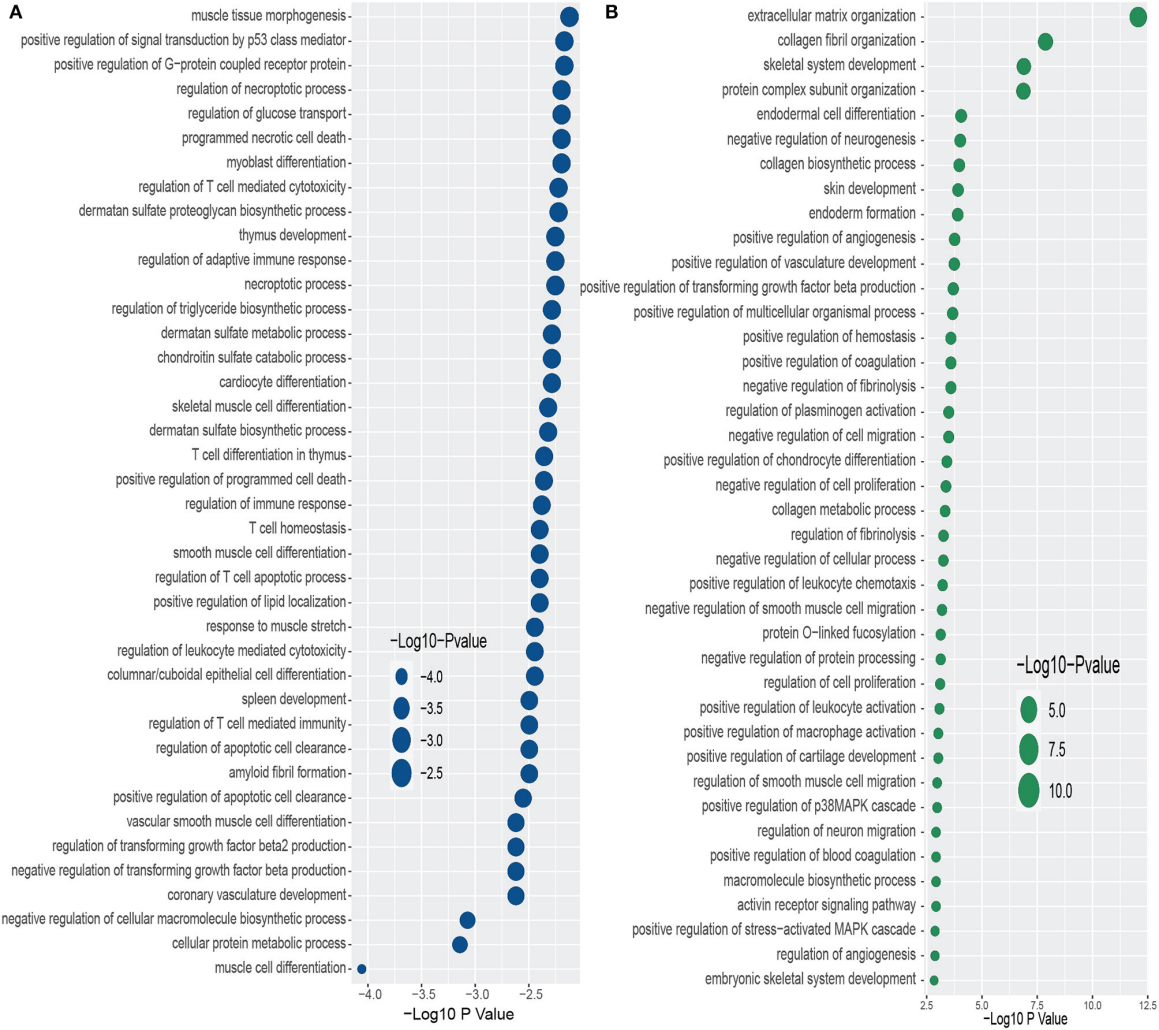
Top 40 GO terms based on the adjusted *p*-values between PD and psychiatric disorders. The GO terms have been identified with the DEGs that are common in psychiatric disorder and Parkinson's disease. **(A, B)** Display the GO pathways for BD and SCZ, respectively.

Furthermore, we have conducted GO term enrichment using the same set of common DEGs between PD and the two psychiatric disorders. For this purpose, We have used the GO biological process (2018) database collected from Enrichr (Kuleshov et al., 2016) libraries. The significantly enriched GO terms are identified if the enrichment yields high logarithmic value of *adjusted p-value*. Figure 4 displays the top 40 GO pathways of PD in relevance to psychiatric disorders. As we can see from Figure 4A, there are several equally significant GO terms are associated with the DEGs. Our investigation reveals several highly enriched GO terms such as muscle tissue morphogenesis, positive regulation of signal transduction by p53 class mediator, positive regulation of G–protein coupled receptor protein, regulation of necroptotic process, regulation of glucose transport, programmed necrotic cell death, to name a few. We have found diverse T cell related and sulfate related GO terms in BD in presence of PD. We have also observed various GO terms which are related to cardiovascular system such as cardiocyte differentiation, coronary vasculature development, vascular smooth muscle cell differentiation, to name a few. Extracellular matrix organization is the highly enriched GO pathway in PD-SCZ interaction (Figure 4B). Collagen fibril organization, Skeletal system development, and Protein complex subunit organization are also strongly enriched GO terms that we found in PD patients' samples in the presence of SCZ. Similar to the PD-BD interaction, we have observed biosynthetic process, vasculature development, and smooth muscle cell related GO terms in PD-SCZ interaction. We have also discovered angiogenesis and O−linked relevant GO terms in SCZ patients with PD conditions.

### 3.4. Gene regulatory network analysis identifies networks of DEGs-miRNA and TF-gene interactions

**Figure 5:**
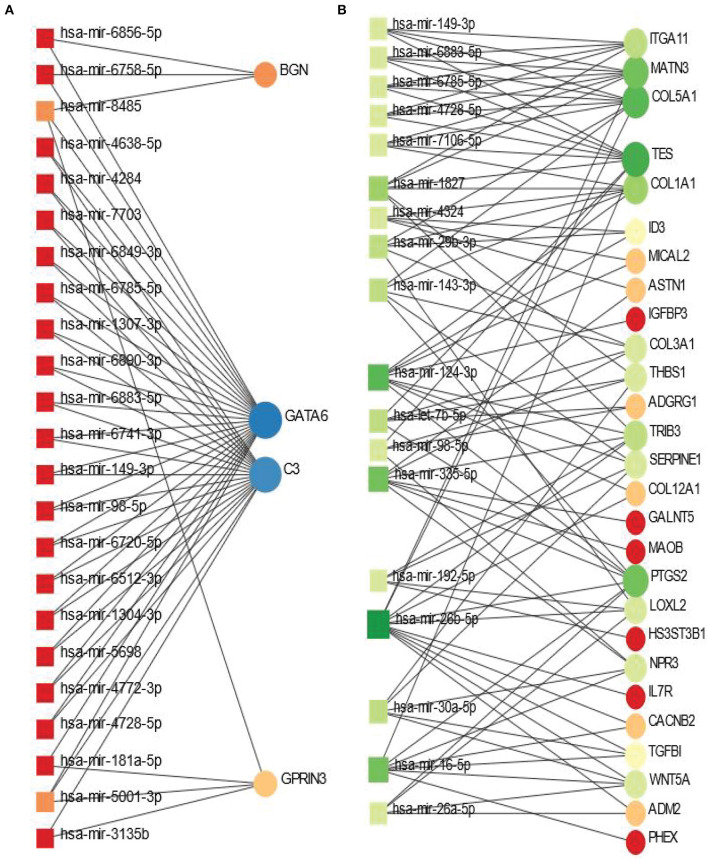
Differentially expressed genes-micro RNA (DEGs-miRNA) networks of **(A)** PD and BD and **(B)** PD and SCZ The networks are filtered with the betweenness value of 500. The circles represent DEGs and the squares represent miRNA. The variations in size and color are used to represent expressed DEGs of different levels.

The shared DEGs between PD and psychiatric disorders have been used in this analysis. The DEG-miRNA interactions network is displayed in Figure 5. We have obtained the networks from Network Analyst (Xia and Hancock, 2015). The circles in the figure represent the dysregulated genes, and the squares represent the miRNAs. Lines connecting the nodes (circles or squares) of the networks represent the association among the nodes. Nodes in a network that connects several edges are termed more significant nodes than others. For example, the network representing the DEG-miRNA association between PD and BD is displayed in Figure 5A. We used the betweenness parameter of 500. In this figure, GATA6 and C3 are two most significant genes as these two genes connect several miRNAs. BGN and GPRIN3 are two other notable DGEs in PD-BD relationship. As far as miRNA is concerned, hsa-mir-8485 and hsa-mir-5001-3p are more highly enriched than others as we see from Figure 5A. In the case of PD and SCZ interaction, The DEGs-miRNA network is displayed in Figure 5B. The dark green circles represent highly enriched DEGs. For example, TES, COL5A1, MATN3, and PTGS2 are heavily expressed DEGs in this context. The genes presented in red circles are relatively less significant since they have only one connection with miRNA. Other genes which show several connections with miRNA include ITGA11, COL1A1, TRIB3 to name a few. The dark brown squares represent significant miRNAs such as hsa-mir-7-5p, hsa-mir-30a-5p, hsa-mir-335-5p.

**Figure 6:**
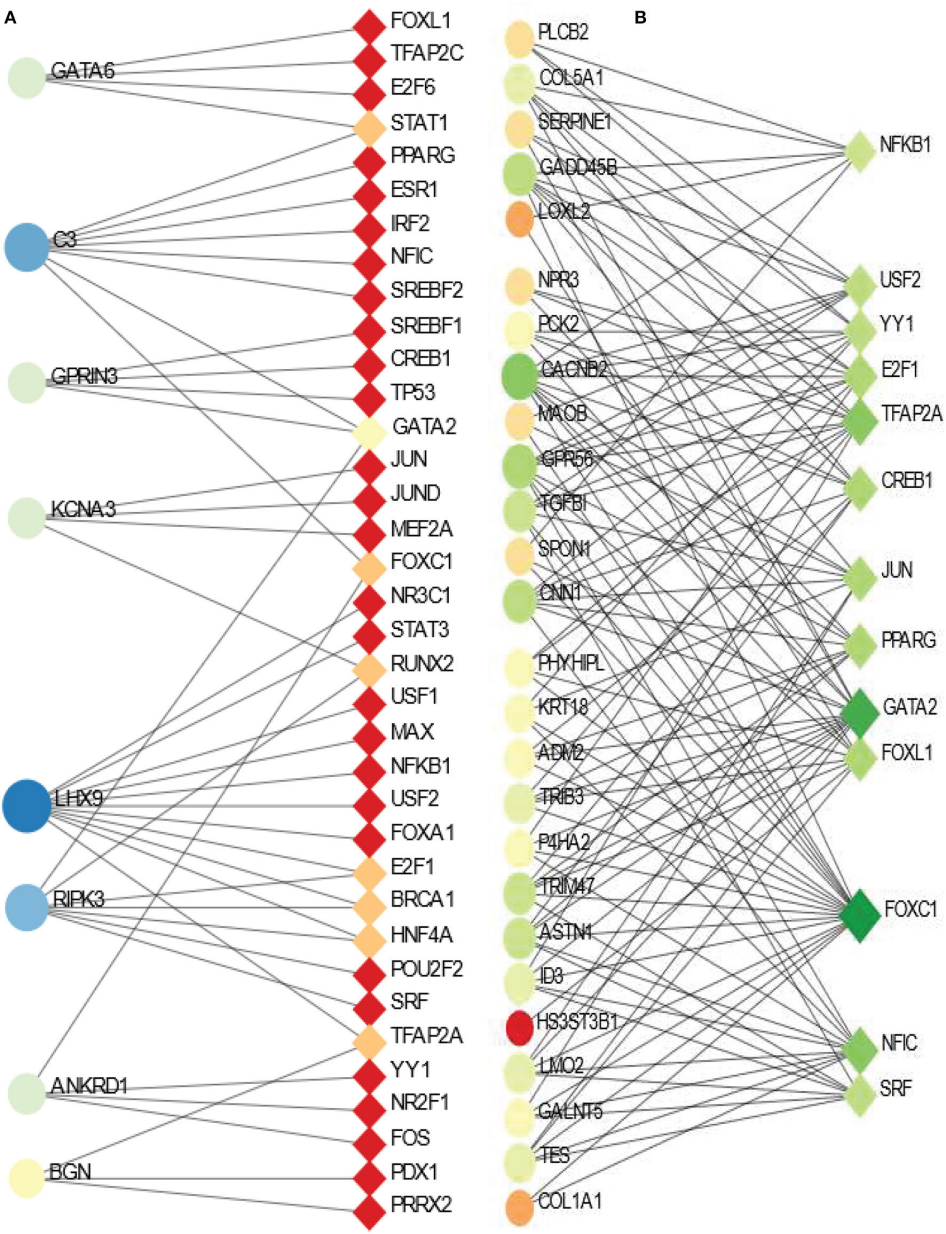
Association between PD and psychiatric disorders from the perspective of dysregulated gene and transcription factors. **(A, B)** Displays the tf-gene networks of PD with BD and SCZ, respectively. The circles represent DEGs and the squares represent transcription factors. The variations in size and color are used to represent expressed DEGs of different levels.

We have also identified transcription factor (TF) - DEGs interaction networks of BD and SCZ with PD. The TF-DEGs networks are displayed in Figure 6. Circles and squares in the figure represent DEGs and TFs respectively. Figure 6A displ ays TF-DEGs network between PD and BD. In this case, we set the betweenness parameter of 500, and the degree parameter of 3 in Network Analyst. As it is seen the most significant DEG in this network is LHX9 which has highest number of edges connected with TFs. Other significant DEGs in this network include C3, RIPK3, GATA6, GPRIN3, KCNA3, ANKRD1. Significant transcription factors in PD-BD interaction include GATA2, STAT1, FOXC1, RUNX2, E2F1, BRCA1, HNF4A, TFAP2A. Figure 6B shows the TF-DEGs network between PD and SCZ. We set the betweenness parameter of 100 in Network Analysis in this analysis. The highly expressed gene in this network includes CACNB2, GPR56, CNN1, GADD45B to name a few. GATA2, FOXC1, NFIC, TFAP2A are among significant transcription factors as we observe from Figure 6B.

### 3.5. Protein-chemical compounds analysis identifies interactions between protein and chemical agents

**Figure 7:**
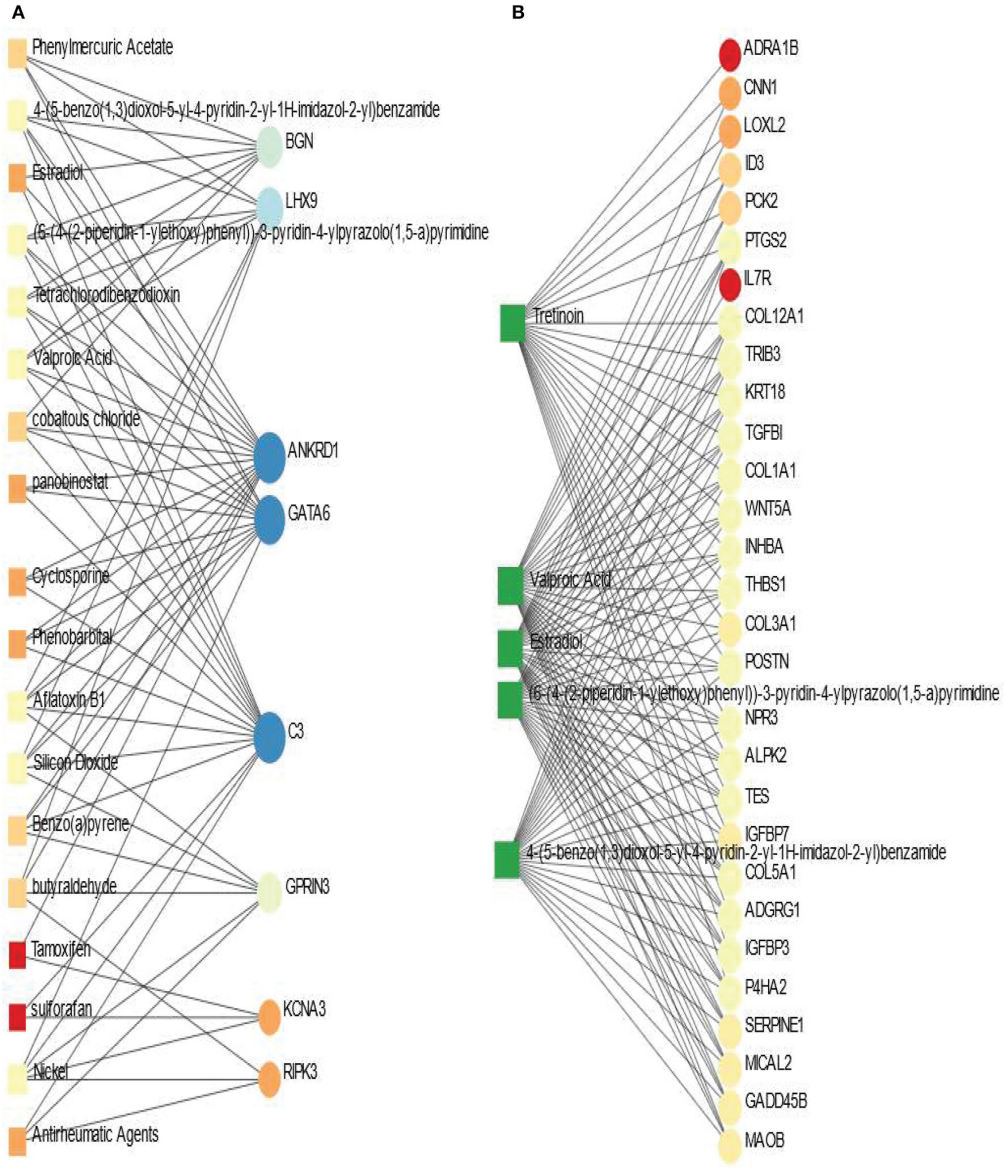
Association between PD and psychiatric disorders from the perspective of protein and chemical agents. **(A, B)** Displays the protein-chemical networks of PD with BD and SCZ, respectively. The circles represent proteins and the squares represent chemical compounds. The variations in size and color are used to represent expressed DEGs of different levels. The squares having several edges are most expressed chemical agents.

Protein-chemical interaction is an important study that plays a significant role in the process of drug discovery. We have discovered the protein-chemical interaction networks of BD and SCZ with PD. The protein-chemical interaction networks are presented in Figure 7. Circles and squares in the figure represent proteins and chemical agents respectively. We have utilized Network Analyst (Xia and Hancock, 2015) to discover the networks in this analysis. Figure 7A displays the protein-chemical interaction network between PD and BD. In this analysis, we have used the betweenness of 200 as a parameter do obtain the network from Network Analyst. The dark blue circles represent the highly expressed proteins in this network which are ANKRD1, GATA6, C3. Other than these proteins BGN, LHX9, GPRIN3 are also notable. As far as chemical agents are concerned, Aflatoxin B1, Nickel, Valproic acid, Silicon Dioxide are some of the highly enriched as observed. Figure 7B shows the protein-chemical network between SCZ and PD. In this case, we have used the betweenness of 500 and the degree filter of 30 in Network Analyst. The highly expressed proteins in this interaction are represented in light colored circles. The highly expressed proteins in this network PTGS2, COL12A1, TRIB3, COL1A1, TES, COL5A1, POSTN, to name a few. As we can see from this network, the important chemical components are Valporic acid, Tretinoin, Estradiol.

## 4. Discussion

This work investigates the influences of PD on patients with psychiatric disorders. We have considered two psychiatric disorders along with PD, and determined the DEGs from each of the datasets representing these diseases. We have identified the overlapping DEGs between each of the psychiatric disorders and PD. In this study, we have found eight overlapping DEGs between BD and PD. The heatmaps in Figures 1E, F show the relationship between PD and BD with respect to these DEGs. We have found GATA6 (GATA binding protein 6), which belongs to the family of zinc finger transcription factors. GATA6 plays a significant role in the regulation of cellular differentiation and organogenesis during vertebrate, heart and lung development (Safran et al., 2010; Sharma et al., 2020). In Jiao et al. (2021), the authors found that GATA6 is a critical regulator increased in aged mesenchymal stem cells (MSCs) that control aging related activities. We have also identified LHX9 (LIM Homeobox 9), which is a protein coding gene, and one study (Hook et al., 2018) found LHX9 a strong candidate gene in their analysis of single cell RNA-seq on sporadic Parkinson's disease. In addition, our result identifies the presence of GPRIN3 (GPRIN Family Member 3). Karadurmus et. al., identifies the association of GPRIN3 in the functionalities of striatum (Karadurmus et al., 2019). The striatum is the basal ganglia's principal input nucleus which integrates sensory, affective, and higher cognitive responses (Maltese et al., 2018). The outcome of the study indicates a prevalent role of GPRIN3 in dopamine signaling in the striatum which can be linked with striatal dysfunction associated with the dopamine receptor, such as Parkinson's disease and psychiatric disorders (Karadurmus et al., 2019). Our results also indicate the existence of RIPK3 (Receptor Interacting Protein Kinases 3) in PD-BD interaction. Evidence suggests that RIPK3 is one of the key components in the process of cell death in brains (Wehn et al., 2021). Patients with brain damage often suffer from neurocognitive and mood disorders, neurocognitive dysfunction, or dementia (Andelic et al., 2018). Other candidate genes found in our result and their biological significance have been presented in later part of the discussion. We have identified 55 genes that are common between PD and SCZ. We have discovered genes of several types from Collagen family shared between PD and SCZ such as COL1A1, COL3A1, COL5A1, COL12A1. Many disorders are caused by mutations in the genes coding for collagen (Pinnell et al., 1972; Ricard-Blum, 2011). In one study (Su et al., 2017), two different datasets were considered, and Collagen genes were included in numerous pathways in both datasets, including abnormal cell adhesion and extracellular matrix in schizophrenia. The finding of our experiment supports the claim made in Su et al. (2017) since our investigation also reveals the Collagen types genes. Jin et al. found compound COL6A3 variants contributed to increase risk of developing PD (Jin et al., 2021). In Merico et al. (2015), the whole genome sequencing identified COL3A1 as one of the genes linked to the development of SCZ. COL3A1 gene was identified as one of the highly expressed genes in the functional gene networks involved in SCZ in another study (Gilman et al., 2012). In addition, COL3A1 is one of the several genes which is present in the gene-disease association dataset presented in Rouillard et al. (2016). Katrina Tiklova et. al., identified the presence of COL1A1 and COL3A1 in PD patients in their study of single cell RNA sequencing on stem cells and ventral midbrain tissue (Tiklová et al., 2020). The expression of COL1A1 was found in PD patients samples in several studies (Choi et al., 2017; Sawangareetrakul et al., 2021). The result of our study also reveals a group of Family With Sequence Similarity genes that include FAM65C, FAM134B and FAM181B. Although the functional role of FAM134B (family with sequence similarity 134, member B) is relatively new in human disease, recent studies show that absence or non-functional FAM134B contributed to the development of neuronal disorders (Murphy et al., 2012; Islam et al., 2018).

With these dysregulated genes, we have conducted the PPI network analyses. As it can be seen from Figure 2, BGN (Biglycan) is one of the highly expressed proteins in the interaction between PD and other two disorders. Moreover, as observed from Figures 1D–F, BGN is the only gene that is shared by all the three disorders considered in our study. One of the roles of this gene is to generate the mature protein, which has a significant influence on bone growth, muscle development and regeneration, and collagen fibril assembly in multiple tissues (Safran et al., 2010). The finding of BGN also indicates the potential relationship of Collagen type genes (the presence of these genes in our work has been described in the earlier paragraph) in the interaction of PD with psychiatric disorders. The presence of BGN expression supports several previous studies which show that the association of BGN with a broad range of neurodegenerative events (Meng et al., 2016; Meester et al., 2017; Ying et al., 2018). A meta analysis done on genomic profiling in schizophrenic patients as compared to healthy controls identified BGN as one of the causative gene for SCZ development (Logotheti et al., 2013). Proteins (COLA1, COL3A1, COL5A1, COL12A1) from Collagen family are highly expressed in PD patients with SCZ condition as we see from the PPI network (Figure 2). In addition to Collagen type genes, CTGF, POSTN, THBS1, ITGA11, LOXL2, TGFBI, IGFBP3, IGFBP7, SERPINE1 are some of the heavily enriched proteins in the interaction between PD and SCZ. Studies show the association of THBS1 (Thrombospondin 1) variants with SCZ and other neurodevelopmental diseases (Park et al., 2012; Hofer et al., 2020). The PPI network also discovered CTGF (connective tissue growth factor), a matricellular protein that is highly expressed in the brain of individuals with Alzheimer's disease and PD (McClain et al., 2009; Mann et al., 2017; Yang et al., 2017).

We have also identified significantly enriched GO terms and signaling pathways with the DEGs. These pathways and GO terms can be useful in determining the linkage among genes to identify potential therapeutic interventions. As we see from Figure 3A, Small leucine–rich proteoglycan (SLRP) molecules and Activation of C3 and C5 are the two top significant signaling pathways in PD and BD interaction. SLRP is involved in a variety of biological and pathological processes. SLRPs have significant structural roles within extracellular matrices, and SLRPs are responsible for bone development and functionalities. BGN is one of the genes of SLRP family (Dellett et al., 2012; Chen and Birk, 2013). In our study, we have found BGN as one of the highly expressed genes that is shared by PD, BP and SCZ. The presence of SLRP also indicates the role of Collagen type genes in the interaction between PD and psychiatric disorders since SLRPs are known to bind to various collagens (Nikitovic et al., 2012; Kram et al., 2017). We have found several RIP1/RIPK1 related pathways. The genes involved in this pathway are interlinked with neuro diseases and cell damage (Wang et al., 2018; Liu et al., 2021). We have also observed some pathways in PD and BD interaction that are associated with bone and mental disorders. The pathways include Defective CHSY1 causes TPBS, Defective CHST3 causes SEDCJD, Defective CHST14 causes EDS, musculocontractural type to name a few (Fabregat et al., 2018). The result of our study also reveals Defective B4GALT7 causes EDS, progeroid type, Defective B3GAT3 causes JDSSDHD, and Defective B3GALT6 causes EDSP2 and SEMDJL1 pathways which are associated with aged appearance, developmental delay, broad spectrum of skeletal, connective tissue and wound healing problems (Fabregat et al., 2018). In the interaction between PD and SCZ, we have observed several TGF-beta related pathways. In our ontology analysis between PD and BD, even though muscle tissue morphogenesis is the most significant GO term, we have observed several T cell related GO terms. In the case of SCZ, Extracellular Matrix Organization is the most significant GO term that we found in our study. Several earlier and recent studies indicate involvement of the brain extracellular matrix (ECM) in the pathophysiology of SCZ. We have also several Collagen related GO term in PD patients with SCZ condition (Berretta, 2012; Pantazopoulos et al., 2021). We have found genes in our study that are that involved in dysregulation of ECM in SCZ patients brain regions. In Pantazopoulos et al. (2021), authors discovered IGFBP3 and group of ADAM genes along with other genes that are key components in dysregulation of ECM. In our study, we have identified IGFBP3 (Insulin Like Growth Factor Binding Protein 3) and ADAMTS15 (ADAM Metallopeptidase With Thrombospondin Type 1 Motif 15) in the association of SCZ with PD. ADAMTS15 is one of the members of ADAMTS family, and plays a significant role in the process of skeletal muscle development (Safran et al., 2010). Thus the finding of our study also supports the growing evidence that dysregulation of ECM may contribute to the development of SCZ.

We have discovered DEG-miRNA, TF-DEG and protein- chemical agents interaction networks in order to understand the significance of the DEGs shared between PD and psychiatric disorders. In the case of BD, the most expressed DEGs in these networks include GATA6 and C3. We have identified hsa-miR-5001-3p and hsa-mir-8485 as two mostly linked miRNA while hsa-mir-5001-3p is associated with both GATA6 and C3. Earlier research found expression of GATA8 in aging, neurodegenerative, and depression disorders (Scherzer et al., 2008; Scarpa et al., 2018; Jiao et al., 2021). In the case of SCZ, we have found COL5A1 as the most significant DEGs that have maximum interaction with different miRNA. In one study (Huang et al., 2021), critical genes were identified from multiple brain regions in PD patients' sample, and the study identified CTGF and COL5A1 are highly expressed in cingulate gyrus region. Our study also shows the high expression of these two genes in the interaction between PD and SCZ. hsa-mir-265-5p, hsa-mir-124-3p, hsa-mir-335-5p are among highly prevalent miRNA in the association of PD with SCZ.

We have also discovered TF-DEG interaction networks between PD and two psychiatric disorders. TFs usually target host proteins to alter their expression during the development of specific diseases. We have found GATA2, FOXC1 and TFAP2A among highly expressed TFs that are associated with DEGs are shared between PD and psychiatric disorders. FOXC1 belongs to the forkhead family of transcription factors. Mutations in FOXC1 are associated with cerebellar malformation and cerebral small vessel disease (Norden et al., 2020). Earlier studies found altered expression of GATA2 in both PD and SCZ patients (Scherzer et al., 2008; Miller et al., 2012). We have observed LHX9, C3, RIPK3, GATA6 are among highly expressed DEGs that are associated with several TFs in the interaction between PD and BD. Hypocretin, also known as Orexin, is a neuropeptide that regulates the normal regulation of sleep and wake behaviors. The absence of these neurons causes narcolepsy in humans and model organisms. Earlier studies found the relation of LHX9 with these neurons that are relevant to psychiatric disorders (Dalal et al., 2013; Sall et al., 2021).

We have also discovered protein-chemical agents or drug compound modules interaction networks using the shared DEGs between the three disorders. We have found some chemical components such as alporic acid, Tretinoin, Estradiol, (6-(4-(2-piperidin-1-yl ethoxy)phenyl))-3-pyridin-4-yl pyrazolo(1,5-a)pyrimidine, 4-(5-benzo(1,3)dioxol-5-yl-4-pyridin-2-yl-1H-imidazol-2-yl)benzamide are associated with several proteins in PD and psychiatric disorders. We have also noticed that Tretinoin also has strong connectivity with many proteins in the case of SCZ and PD interaction. In protein-chemical agents interaction analysis, C3, GATA6, ANKRD1, LHX9, BGN are some of the highly expressed proteins in PD and BD combinations. These proteins were also expressed highly in our previous analysis.

## 5. Conclusion

Overall, this research concentrates on the molecular understanding of potential biomarkers, regulatory elements, and molecular checkpoints that may help in the development of therapies to treat PD in conjunction with psychiatric illnesses.

The result of this study has discovered several significant genes. Based on gene expression analysis, we highlight significantly expressed genes associated with PD in regards to diseasome and comorbidities. The identified biomarkers could be potential drug targets based on their role in regulatory checkpoints and disease progression. Our observation reveals that the number of shared genes between SCZ and PD is greater than the number of common genes between PD and BD. We have provided gene expression analysis with the identified biomarkers to discover the cell signaling and gene ontology pathways. In addition, we have performed PPIs to identify the significant proteins and their relationships in PD with the presence of psychiatric disorders. Moreover, the GRN analysis identifies the involvement of potential TFs and miRNA, which highlights the risk factors associated with PD. The analysis of the interaction between proteins and existing drug compounds reveals the presence of significant active chemical agents. Furthermore, the identified protein chemical agents and the TFs could be used to target-specific disease-modifying factors. The results of this investigation may offer important new therapeutic approaches to treat PD patients with psychiatric disorders.

## Data availability statement

Publicly available datasets were analyzed in this study. This data can be found here: https://www.ncbi.nlm.nih.gov/geo/.

## Author contributions

MN conceived and designed the study, conducted the experiments, performed the computational analysis, prepared the illustrations, and wrote the manuscript. SS contributed in computational analysis and manuscript writing. SA contributed in critical revision. MM supervised the whole study. All authors approved the final version for submission.
